# Great apes reach momentary altered mental states by spinning

**DOI:** 10.1007/s10329-023-01056-x

**Published:** 2023-03-14

**Authors:** Adriano R. Lameira, Marcus Perlman

**Affiliations:** 1 Department of Psychology, University of Warwick, Coventry, UK; 2 Department of English Language and Linguistics, University of Birmingham, Birmingham, UK

**Keywords:** Altered mental states, Consciousness, Great apes, Human evolution, Origins of the mind, Spinning

## Abstract

Among animals, humans stand out in their consummate propensity to self-induce altered states of mind. Archaeology, history and ethnography show these activities have taken place since the beginnings of civilization, yet their role in the emergence and evolution of the human mind itself remains debatable. The means through which modern humans actively alter their experience of self and reality frequently depend on psychoactive substances, but it is uncertain whether psychedelics or other drugs were part of the ecology or culture of pre-human ancestors. Moreover, (nonhuman) great apes in captivity are currently being retired from medical research, rendering comparative approaches thus far impracticable. Here, we circumvent this limitation by harnessing the breadth of publicly available YouTube data to show that apes engage in rope spinning during solitary play. When spinning, the apes achieved speeds sufficient to alter self-perception and situational awareness that were comparable to those tapped for transcendent experiences in humans (e.g. Sufi whirling), and the number of revolutions spun predicted behavioural evidence for dizziness. Thus, spinning serves as a self-sufficient means of changing body-mind responsiveness in hominids. A proclivity for such experiences is shared between humans and great apes, and provides an entry point for the comparative study of the mechanisms, functions, and adaptive value of altered states of mind in human evolution.

**Supplementary Information:**

The online version contains supplementary material available at 10.1007/s10329-023-01056-x.

## Introduction

Seeking altered mental states is seemingly a human universal, historically and culturally (Slingerland 2021; Biwer et al. 2022). The biological and behavioural precursors of such experiences are, however, unclear (MacKenna 1992; Pollan 2019), notably because it is challenging to confidently confirm if substance use was viable within the highly diversified ecological and cultural human paleobackdrop (Bergström et al. 2021). Whether altered state experiences within the hominid family shaped the emergence and evolution of the modern human mind remains one of the major and most thought-provoking unknowns in cognitive science.

Wild primates consume fermented foods with alcoholic content (reviewed in Hockings et al. 2015; Amato et al. 2021). Consumption of these foods typically depends on natural availability and the opportunistic use of scarce resources (Hockings et al. 2015). Though consumption of fermented foods by primates suggests that they may indeed experience “drunkenness”, consumption may be primarily driven by the high caloric content of alcohol, and thus a side-effect and “unavoidable consequence of frugivory” (Hockings et al. 2015). Medical research has also focused on drug- and alcohol-related behaviours in primates (Grant and Bennett 2003; Wakeford et al. 2018), but has primarily focused on substance abuse and addiction, where subjects are externally administered these substances. Evolutionary interpretation is, thus, tenuous at best. Here, we propose and explore the use of a new comparative behavioural model for the study of proactive divergence from normal waking states in human evolution: the act of spinning.

Apart from sensing sound (Ghazanfar and Hauser 2001), the vertebrate inner ear monitors and senses changes in body motion, orientation, position, and velocity (Lowenstein 1948). Spinning, i.e. rapidly rotating around one’s body axis, mechanically disrupts inner ear homeostasis and sends nerve signals to the brain that conflict with those from automatic eye movements (Nigmatullina et al. 2015). In humans, this neuronal cross-signalling prompts the perception of a whirling world, along with dizziness, light-headedness, head rushes, vertigo, elation, and other altered states of perception, mood, and consciousness. Averting these symptoms when spinning at high or sustained speeds requires extensive dedicated training (e.g. professional dancers, circus performers, astronauts). More generally, in untrained neurotypical humans, spinning is proactively tapped for rapture (e.g. by means of playground spinner bowls, merry-go-rounds, carousels) and collective or spiritual experiences (e.g. by Sufi whirling dervishes). In autistic children and adults, spinning is also used as a self-stimulating behaviour to control sensory input or intentionally block out external input (British National Autistic Society). Homology in inner ear anatomy among hominids (Quam et al. 2015; Braga et al. 2017) and relatively similar body size (among primates) suggest that similar spinning behaviours may engender similar neurophysiological effects in nonhuman great apes (hereafter, ‘great apes’) and humans. Thus, ancestral individuals, irrespective of whether they had access to psychotropic drugs, may have had the ability to self-induce altered states of proprioception and awareness by spinning.

## Methods

To test and provide a proof of concept for the assumption that spinning induces altered mental states in great apes, and potentially did so as well in human ancestors, we searched YouTube (Alphabet Inc.) for publicly posted and publicly available videos of great apes spinning. We used image-based measures to quantify rotational speeds and rotational duration (see Supplementary Methods). We focused on rope spinning (Byrne et al. 2017), which in our sample mainly occurred as solitary play without evident causes or goals beyond the act itself. Ropes—or rope-like items like vines—likely enable apes to achieve faster free rotations and longer rotation lengths, allowing us to explore the physiological and motoric limits that great apes can experience autonomously. Because the ear and eye anatomy underlying the neurophysiology of spinning are fundamentally the same across body sizes of the same species, we had no prior expectations about differences in rotational speed, rotational length or their respective effects between, for instance, adolescents and adults or males and females.

We then compared ape spinning speeds with expert human spinning in different dance and traditional styles, namely, self-revolving pirouettes performed by professional ballet and Ukrainian hopak dancers, Sufi whirling dervishes, and suspended spinning rope acts by circus artists. These acts require extensive training and dedicated practice to suppress the neurophysiological effects associated with rapid and/or prolonged spinning that untrained individuals would normally suffer. In the case of Sufi whirling, spinning is deliberately used to induce states of mystic experience and spiritual trance. We used these cultural traditions from around the world as baselines for rotational speeds that can be confidently assumed to significantly disrupt the normal waking state in humans and, due to biological and behavioural homology and similar body size, in great apes.

## Results

**Fig. 1A–E:**
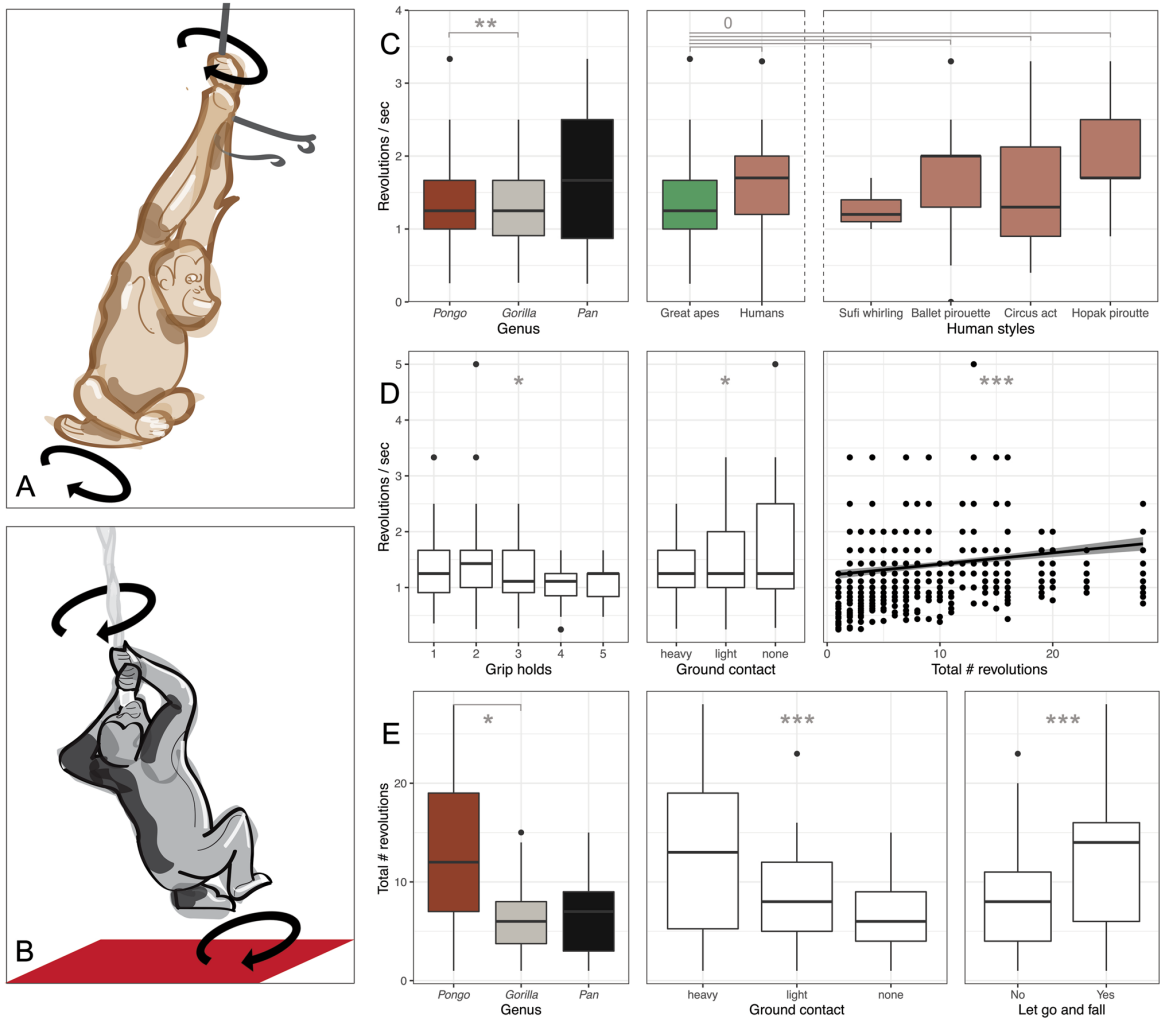
Great ape spinning compared. **A** and **B**: Exemplar cases of rope spinning by an orangutan and a gorilla, respectively. **C:** Comparison of rotational speed between great ape genera, between apes and humans, and between apes and traditional human styles. **D:** Behavioural correlates of rotational speed between great apes. **E:** Number of revolutions between great apes and their behavioural correlates.* Box plots* represent the median (*thick horizontal line*) and 25–75% interquartile range (*upper* and* lower horizontal lines*);* whiskers* represent lowest/highest value within 1.5 times the interquartile range;* dots* represent outliers. Graphic representations based on raw data; denoted differences based on model estimates. * *P* < 0.05, ** *P* < 0.01, *** *P* < 0.001,* zero* no statistically significant difference

We uncovered 40 videos containing footage of great apes engaged in 132 bouts of rope spinning, for a total of 709 revolutions (Fig. 1A, B; see videos in Supplementary Methods). These videos included footage of orangutans from at least seven different identified sites (plus four unidentified sites); gorillas from at least seven identified sites, including two identified sites in the wild (plus three unidentified sites); chimpanzees from two identified sites; and bonobos from four identified sites. Great apes spun, on average, 5.4 revolutions per bout, for an average of 3.3 consecutive spinning bouts, at an average rotational velocity of 1.43 revolutions/second (rps). The longest bout was 28 revolutions; the fastest sustained rotational speed (for five spins) was 3.3 rps; and the fastest single revolution was 5 rps; all of which clearly indicated that spinning is not an “erratic” behaviour.

Linear mixed modelling (see Supplementary Methods) indicated that rotational velocity was proportional to revolution count [linear mixed model (LMM) analysis of variance, *F*_(1, 102.34)_ = 31.101, *P* < 0.001], with higher speeds being achieved with more revolutions (Fig. 1D). There were differences in rotational speed depending on contact with the ground [*F*_(2, 133.65)_ = 4.003, *P* = 0.02] and the number of grip holds during spinning [*F*_(1, 136.62)_ = 4.783, *P* = 0.03], with specific types of grip allowing longer spins (Fig. 1D), tentatively because certain positions may afford different moments of inertia. There was a significant effect of genus [*F*_(2, 38.92)_ = 7.761, *P* = 0.001], with orangutans spinning at significantly faster speeds than gorillas (*t* =  – 3.082, *P* = 0.004; Fig. 1C).

For comparison, an LMM that included data on humans (revolutions, *n* = 1157, *n*_bouts_ = 152, *n*_videos_ = 48) did not indicate differences in rotational speed, either between great apes and humans [*F*_(1, 41.30)_ = 3.355, *P* = 0.074] or between great apes and each specific traditional style [*F*_(4, 36.69)_ = 1.949, *P* = 0.123] (Fig. 1C).

Total number of revolutions in the great apes was influenced by their contact with the ground [*F*_(2, 700.57)_ = 23.516, *P* < 0.001], with more support leveraging more revolutions (Fig. 1E). There was a strong trend for genus [*F*_(2, 37.99)_ = 3.163, *P* = 0.054], with orangutans spinning for more revolutions per bout than gorillas [*t* = 2.312, *P* = 0.026; Fig. 1E]. Crucially, when apes spun for a higher number of revolutions, they were more likely to let go of the rope or let it go slack at the end of the bout [*F*_(1, 693.25)_ = 81.733, *P* < 0.001], suggesting that they experienced dizziness. Closer inspection of the 43 cases when individuals released the rope revealed further evidence of dizziness: in 30 of the bouts, the animal immediately sat or laid down; in seven of the bouts, the animal moved a short distance and then sat or laid down; and in only six bouts did the animal keep its balance and remain on its feet.

## Discussion

Our findings show that great apes spin at speeds that induce physiological “highs” in humans. In untrained humans, spinning at similar rates inescapably produces severe dizziness (we invite the reader to try the observed average rotational speed, length or number of bouts performed by great apes reported here for instant validation). Notably, by comparing “recreational” spinning behaviour of apes to professional spinning in humans, our analyses were inherently conservative. Our findings, while exploratory, provide a proof of concept and a new charter for the study and comparison of spinning and altered mental states between humans and great apes.

Our preliminary findings point to several directions for the future study of spinning behaviour in apes and other species. One is to investigate questions related to evo-ecological constraints on spinning. For example, differences in spinning between orangutans (which are mostly arboreal) and gorillas (which are mostly ground-dwelling) could suggest neurological adaptation against motion sickness or vertigo (with faster speeds/more revolutions required for arboreal species to reach dizziness), similar to the reduction of the vestibular cerebellum observed in ballerinas and figure skaters (Nigmatullina et al. 2015). Differences in certain anatomical features between species may also help them leverage more or fewer revolutions when spinning (e.g. the gorillas never used foot grips, while the orangutans often did).

Our findings also raise interesting questions concerning whether this behaviour is performed more frequently by a particular age class or sex, for example, as part of play by juveniles or as part of male display. Because this behaviour in great apes appears to be idiosyncratic, performed by certain individuals rather than occurring across populations, we anticipate that answering these questions will pose an empirical challenge. If attainable, such effort could help provide new insight into the motivation for spinning behaviour and its ontogeny.

More conclusive comparisons between species, as well as between age classes and sexes, could be made possible by controlling for the proportions in which the relevant groups occur in captivity. For example, our findings suggest that bonobos—who are relatively scarce in captivity compared to chimpanzees, but were conversely well represented in our dataset—may more frequently engage in rope spinning than their sister species. Unexpectedly high rates of occurrence in a species with relatively small population sizes in captivity could suggest a higher predisposition to engaging in behaviour that leads to altered states.

Comparisons of captive and wild populations based on more data could also inform whether this behaviour is more likely to occur in animals in captivity, where they might engage in spinning and experience the ensuing state of dizziness as a way of overcoming low environmental stimulation or boredom. However, comparisons using data on wild individuals will probably be limited because recordings of them are rare and idiosyncratic (e.g. footage of wild mountain gorillas was present in our data, though this was the result of video coverage of some gorilla groups as a consequence of tourists filming them).

Although beyond the scope of analysis here, we have also observed videos of rope spinning by other primate species, including gibbons and monkeys. Future research may seek to determine whether other primates spin as frequently as great apes and in such a way that elicits dizziness and altered mental states. Increasing phylogenetic distance will, however, reduce interpretative power based on the physiological and cognitive homology of these species with humans.

To establish clear comparative benchmarks for future ape-human comparisons, it would be relevant to determine the minimum spinning speeds and lengths of time engaged in spinning necessary to induce altered states in humans, and how training affects and extends these limits. Ethnographic and anthropological studies of how children and adults use spinning and other non-pharmacological means to deliberately disrupt body and situational awareness (e.g. swings, slides, rollercoasters, bungee jumping) could provide complementary information about the role that these experiences play in our lives and, by extension, those of our ancestors over evolutionary time. Interestingly, some accredited zoos are reported to have re-used equipment from children’s playgrounds as enclosure enrichment for apes (R. Shumaker, personal communication). Widespread adoption of devices that make up typical children’s playgrounds for use in great ape facilities could provide dynamic stimulation and motoric challenge to individuals, while potentially helping to reveal more comprehensively, and in a controlled fashion, how and why great apes engage in mind-altering behaviours.

### Concluding remarks

The findings reported here show that, like humans, great apes voluntarily seek and engage in altered experiences of self-perception and situational awareness. In our last common ancestors, these behaviours probably enhanced the nervous system and musculature (Byrne 2015), which helped to expand the range of action patterns, but also momentarily altered the inner world, range and patterns of perception, emotions, and (self- and other-) awareness of these individuals. The empirical evidence presented here provides some grounding for the intriguing possibility that the self-induced altered mental states of our ancestors could have shaped aspects of modern human behaviour and cognition, as well as mood manipulation and mental wellbeing.

## Supplementary Information

Below is the link to the electronic supplementary material.

Supplementary file1 (DOCX 31 KB)

Supplementary file2 (DOCX 24 KB)

## Data Availability

All data needed to evaluate the conclusions in the paper are present in the paper and/or the Supplementary Materials. Additional data related to this paper may be requested from the authors.

## References

[CR1] Amato KR, Chaves ÓM, Mallott EK (2021). Fermented food consumption in wild nonhuman primates and its ecological drivers. Am J Phys Anthropol.

[CR2] Bergström A, Stringer C, Hajdinjak M (2021). Origins of modern human ancestry. Nature.

[CR3] Biwer ME, Álvarez WY, Bautista SL, Jennings J (2022). Hallucinogens, alcohol and shifting leadership strategies in the ancient Peruvian Andes. Antiquity.

[CR4] Braga J, Bouvier P, Dherbey J (2017). Echoes from the past: new insights into the early hominin cochlea from a phylo-morphometric approach. CR Palevol.

[CR5] Byrne RW (2015). The what as well as the why of animal fun. Curr Biol.

[CR6] Byrne R, Cartmill E, Genty E (2017). Great ape gestures: intentional communication with a rich set of innate signals. Anim Cogn.

[CR7] Ghazanfar AA, Hauser MD (2001). The auditory behaviour of primates: a neuroethological perspective. Curr Opin Neurobiol.

[CR8] Grant KA, Bennett AJ (2003). Advances in nonhuman primate alcohol abuse and alcoholism research. Pharmacol Ther.

[CR9] Hockings KJ, Nicola B-M, Carvalho S (2015). Tools to tipple: ethanol ingestion by wild chimpanzees using leaf-sponges. Royal Soc Open Sci.

[CR10] Lowenstein O (1948). Oscillographic analysis of the non-acoustic functions of the vertebrate ear. Nature.

[CR11] MacKenna T (1992). Food of the gods: the search for the original tree of knowledge: a radical history of plants, drugs, and human evolution.

[CR12] Nigmatullina Y, Hellyer PJ, Nachev P (2015). The neuroanatomical correlates of training-related perceptuo-reflex uncoupling in dancers. Cereb Cortex.

[CR13] Pollan M (2019). How to change your mind: the new science of psychedelics.

[CR14] Quam R, Martínez I, Rosa M (2015). Early hominin auditory capacities. Sci Adv.

[CR15] Slingerland E (2021). Drunk: how we sipped, danced, and stumbled our way to civilization.

[CR16] Wakeford AGP, Morin EL, Bramlett SN (2018). A review of nonhuman primate models of early life stress and adolescent drug abuse. Neurobiol Stress.

