# Supplementary material for: Great apes reach momentary altered mental states by spinning
Source: Primates. 2023 Mar 14;64(3):319–23. doi: 10.1007/s10329-023-01056-x (PMC10185630; doi:10.1007/s10329-023-01056-x)
Supplement: Supplementary file 1 — Supplementary file1 (DOCX 31 KB) [file 10329_2023_1056_MOESM1_ESM.docx]

**Great apes reach momentary altered mental states by spinning**

Adriano R. Lameira^1*^, Marcus Perlman^2*^

^1^Department of Psychology, University of Warwick, UK

^2^Department of English Language and Linguistics, University of Birmingham, UK

^*^Corresponding author: [adriano.lameira@warwick.ac.uk](mailto:adriano.lameira@warwick.ac.uk), [m.perlman@bham.ac.uk](mailto:m.perlman@bham.ac.uk)

**Supplementary Data**

*Linear Mixed Models: Revolutions per second (Apes only)*

| **ANOVA Summary** | | | | | | | |
| --- | --- | --- | --- | --- | --- | --- | --- |
| **Effect** | | **df** | | **F** | | **p** | |
| Ground |  | 2, 133.65 |  | 4.003 |  | 0.020 |  |
| Let go rope |  | 1, 111.02 |  | 0.001 |  | 0.971 |  |
| Bout total Rev |  | 1, 102.34 |  | 31.101 |  | < .001 |  |
| Grip |  | 1, 136.62 |  | 4.783 |  | 0.030 |  |
| Genus |  | 2, 38.92 |  | 7.761 |  | 0.001 |  |
|  | | | | | | | |
| *Note.*  Model terms tested with Satterthwaite method. | | | | | | | |
| *Note.*  The following variables are used as random effects grouping factors: 'VIDEO', 'BOUT'. | | | | | | | |
| *Note.*  Type III Sum of Squares | | | | | | | |

**Model summary**

| **Fit statistics** | | | | | | | | | |
| --- | --- | --- | --- | --- | --- | --- | --- | --- | --- |
| **Deviance (REML)** | | **log Lik.** | | **df** | | **AIC** | | **BIC** | |
| 959.059 |  | -479.530 |  | 11 |  | 981.059 |  | 1031.261 |  |
|  | | | | | | | | | |
| *Note.*  The model was fitted using restricted maximum likelihood. Please note that models with different fixed effects cannot be compared when REML is used. To use ML, switch 'Test model terms' to 'Likelihood ratio tests'. | | | | | | | | | |

| **Sample sizes** | | | | | |  |  |  |  |  |  |  |  |
| --- | --- | --- | --- | --- | --- | --- | --- | --- | --- | --- | --- | --- | --- |
|  | | **Levels of RE grouping factors** | | | |  |  |  |  |  |  |  |  |
| **Observations** | | **BOUT** | | **VIDEO** | |  |  |  |  |  |  |  |  |
| 709 |  | 132 |  | 40 |  |  |  |  |  |  |  |  |  |
|  | | | | | |  |  |  |  |  |  |  |  |
| **Fixed Effects Estimates** | | | | | | | | | | | | | |
| **Term** | | | **Estimate** | | | **SE** | | **df** | | **t** | | **p** | |
| Intercept | |  | 1.443 | |  | 0.147 |  | 127.490 |  | 9.837 |  | < .001 |  |
| Ground (1) | |  | 0.068 | |  | 0.059 |  | 136.599 |  | 1.151 |  | 0.252 |  |
| Ground (2) | |  | 0.101 | |  | 0.060 |  | 128.462 |  | 1.677 |  | 0.096 |  |
| Let go rope (1) | |  | -0.001 | |  | 0.040 |  | 111.015 |  | -0.037 |  | 0.971 |  |
| Bout total Rev | |  | 0.045 | |  | 0.008 |  | 102.341 |  | 5.577 |  | < .001 |  |
| Grip | |  | -0.114 | |  | 0.052 |  | 136.624 |  | -2.187 |  | 0.030 |  |
| Genus 1 (1) | |  | -0.276 | |  | 0.090 |  | 38.901 |  | -3.082 |  | 0.004 |  |
| Genus 1 (2) | |  | -0.157 | |  | 0.096 |  | 39.189 |  | -1.634 |  | 0.110 |  |
|  | | | | | | | | | | | | | |

*Note.*  The intercept corresponds to the (unweighted) grand mean; for each factor with k levels, k - 1 parameters are estimated with sum contrast coding. Consequently, the estimates cannot be directly mapped to factor levels. Use estimated marginal means for obtaining estimates for each factor level/design cell or their differences.

## *Linear Mixed Models: Revolutions per second (Apes vs humans)*

| **ANOVA Summary** | | | | | | | |
| --- | --- | --- | --- | --- | --- | --- | --- |
| **Effect** | | **df** | | **F** | | **p** | |
| Clade |  | 1, 41.30 |  | 3.355 |  | 0.074 |  |
|  | | | | | | | |
| Note.  Model terms tested with Satterthwaite method. | | | | | | | |
| Note.  The following variables are used as random effects grouping factors: 'VIDEO', 'BOUT'. | | | | | | | |
| Note.  Type III Sum of Squares | | | | | | | |

### Model summary

| **Fit statistics** | | | | | | | | | |
| --- | --- | --- | --- | --- | --- | --- | --- | --- | --- |
| **Deviance (REML)** | | **log Lik.** | | **df** | | **AIC** | | **BIC** | |
| 1644.049 |  | -822.025 |  | 5 |  | 1654.049 |  | 1679.317 |  |
|  | | | | | | | | | |
| Note.  The model was fitted using restricted maximum likelihood. Please note that models with different fixed effects cannot be compared when REML is used. To use ML, switch 'Test model terms' to 'Likelihood ratio tests'. | | | | | | | | | |

| **Sample sizes** | | | | | |  |  |  |  |  |  |  |
| --- | --- | --- | --- | --- | --- | --- | --- | --- | --- | --- | --- | --- |
|  | | **Levels of RE grouping factors** | | | |  |  |  |  |  |  |  |
| **Observations** | | **BOUT** | | **VIDEO** | |  |  |  |  |  |  |  |
| 1157 |  | 152 |  | 48 |  |  |  |  |  |  |  |  |
|  | | | | | |  |  |  |  |  |  |  |
| **Fixed Effects Estimates** | | | | | | | | | | | | |
| **Term** | | | **Estimate** | | **SE** | | **df** | | **t** | | **p** | |
| Intercept | |  | 1.527 |  | 0.092 |  | 41.303 |  | 16.605 |  | < .001 |  |
| Clade (1) | |  | -0.168 |  | 0.092 |  | 41.303 |  | -1.832 |  | 0.074 |  |
|  | | | | | | | | | | | | |
| Note.  The intercept corresponds to the (unweighted) grand mean; for each factor with k levels, k - 1 parameters are estimated with sum contrast coding. Consequently, the estimates cannot be directly mapped to factor levels. Use estimated marginal means for obtaining estimates for each factor level/design cell or their differences. | | | | | | | | | | | | |

##

## *Linear Mixed Models: Revolutions per second (Ape vs each human tradition)*

| **ANOVA Summary** | | | | | | | |
| --- | --- | --- | --- | --- | --- | --- | --- |
| **Effect** | | **df** | | **F** | | **p** | |
| Clade |  | 4, 36.69 |  | 1.949 |  | 0.123 |  |
|  | | | | | | | |
| Note.  Model terms tested with Satterthwaite method. | | | | | | | |
| Note.  The following variables are used as random effects grouping factors: 'VIDEO', 'BOUT'. | | | | | | | |
| Note.  Type III Sum of Squares | | | | | | | |

### Model summary

| **Fit statistics** | | | | | | | | | |
| --- | --- | --- | --- | --- | --- | --- | --- | --- | --- |
| **Deviance (REML)** | | **log Lik.** | | **df** | | **AIC** | | **BIC** | |
| 1641.127 |  | -820.563 |  | 8 |  | 1657.127 |  | 1697.556 |  |
|  | | | | | | | | | |
| Note.  The model was fitted using restricted maximum likelihood. Please note that models with different fixed effects cannot be compared when REML is used. To use ML, switch 'Test model terms' to 'Likelihood ratio tests'. | | | | | | | | | |

| **Sample sizes** | | | | | |  |  |  |  |  |  |  |
| --- | --- | --- | --- | --- | --- | --- | --- | --- | --- | --- | --- | --- |
|  | | **Levels of RE grouping factors** | | | |  |  |  |  |  |  |  |
| **Observations** | | **BOUT** | | **VIDEO** | |  |  |  |  |  |  |  |
| 1157 |  | 152 |  | 48 |  |  |  |  |  |  |  |  |
|  | | | | | |  |  |  |  |  |  |  |
| **Fixed Effects Estimates** | | | | | | | | | | | | |
| **Term** | | | **Estimate** | | **SE** | | **df** | | **t** | | **p** | |
| Intercept | |  | 1.628 |  | 0.139 |  | 35.633 |  | 11.676 |  | < .001 |  |
| Clade (1) | |  | -0.270 |  | 0.151 |  | 36.158 |  | -1.788 |  | 0.082 |  |
| Clade (2) | |  | 0.263 |  | 0.275 |  | 30.287 |  | 0.957 |  | 0.346 |  |
| Clade (3) | |  | 0.462 |  | 0.288 |  | 37.120 |  | 1.604 |  | 0.117 |  |
| Clade (4) | |  | -0.061 |  | 0.367 |  | 31.451 |  | -0.167 |  | 0.869 |  |
|  | | | | | | | | | | | | |
| Note.  The intercept corresponds to the (unweighted) grand mean; for each factor with k levels, k - 1 parameters are estimated with sum contrast coding. Consequently, the estimates cannot be directly mapped to factor levels. Use estimated marginal means for obtaining estimates for each factor level/design cell or their differences. | | | | | | | | | | | | |

##

## *Linear Mixed Models: Total number of revolutions per bout (Apes only)*

| **ANOVA Summary** | | | | | | | |
| --- | --- | --- | --- | --- | --- | --- | --- |
| **Effect** | | **df** | | **F** | | **p** | |
| Ground |  | 2, 700.57 |  | 23.516 |  | < .001 |  |
| Let go rope |  | 1, 693.25 |  | 81.733 |  | < .001 |  |
| Grip |  | 1, 697.72 |  | 3.314 |  | 0.069 |  |
| Genus |  | 2, 37.99 |  | 3.163 |  | 0.054 |  |
|  | | | | | | | |
| Note.  Model terms tested with Satterthwaite method. | | | | | | | |
| Note.  The following variable is used as a random effects grouping factor: 'VIDEO'. | | | | | | | |
| Note.  Type III Sum of Squares | | | | | | | |

### Model summary

| **Fit statistics** | | | | | | | | | |
| --- | --- | --- | --- | --- | --- | --- | --- | --- | --- |
| **Deviance (REML)** | | **log Lik.** | | **df** | | **AIC** | | **BIC** | |
| 3851.215 |  | -1925.607 |  | 9 |  | 3869.215 |  | 3910.289 |  |
|  | | | | | | | | | |
| Note.  The model was fitted using restricted maximum likelihood. Please note that models with different fixed effects cannot be compared when REML is used. To use ML, switch 'Test model terms' to 'Likelihood ratio tests'. | | | | | | | | | |

| **Sample sizes** | | | | |  |  |  |  |  |  |  |  |
| --- | --- | --- | --- | --- | --- | --- | --- | --- | --- | --- | --- | --- |
|  | | **Levels of RE grouping factors** | | |  |  |  |  |  |  |  |  |
| **Observations** | | **VIDEO** | | |  |  |  |  |  |  |  |  |
| 709 |  | 40 |  | |  |  |  |  |  |  |  |  |
|  | | | | |  |  |  |  |  |  |  |  |
| **Fixed Effects Estimates** | | | | | | | | | | | | |
| **Term** | | | **Estimate** | | **SE** | | **df** | | **t** | | **p** | |
| Intercept | |  | 8.694 |  | 1.077 |  | 79.360 |  | 8.075 |  | < .001 |  |
| Ground (1) | |  | -2.199 |  | 0.321 |  | 700.685 |  | -6.857 |  | < .001 |  |
| Ground (2) | |  | 0.880 |  | 0.332 |  | 701.930 |  | 2.652 |  | 0.008 |  |
| Let go rope (1) | |  | -1.619 |  | 0.179 |  | 693.248 |  | -9.041 |  | < .001 |  |
| Grip | |  | -0.513 |  | 0.282 |  | 697.719 |  | -1.820 |  | 0.069 |  |
| Genus (1) | |  | 2.573 |  | 1.113 |  | 37.451 |  | 2.312 |  | 0.026 |  |
| Genus (2) | |  | -1.775 |  | 1.192 |  | 38.348 |  | -1.489 |  | 0.145 |  |
|  | | | | | | | | | | | | |
| Note.  The intercept corresponds to the (unweighted) grand mean; for each factor with k levels, k - 1 parameters are estimated with sum contrast coding. Consequently, the estimates cannot be directly mapped to factor levels. Use estimated marginal means for obtaining estimates for each factor level/design cell or their differences. | | | | | | | | | | | | |
