# Supplementary material for: Great apes reach momentary altered mental states by spinning
Source: Primates. 2023 Mar 14;64(3):319–23. doi: 10.1007/s10329-023-01056-x (PMC10185630; doi:10.1007/s10329-023-01056-x)
Supplement: Supplementary file 2 — Supplementary file2 (DOCX 24 KB) [file 10329_2023_1056_MOESM2_ESM.docx]

**Great apes reach momentary altered mental states by spinning**

Adriano R. Lameira^1*^, Marcus Perlman^2*^

^1^Department of Psychology, University of Warwick, UK

^2^Department of English Language and Linguistics, University of Birmingham, UK

^*^Corresponding author: [adriano.lameira@warwick.ac.uk](mailto:adriano.lameira@warwick.ac.uk), [m.perlman@bham.ac.uk](mailto:m.perlman@bham.ac.uk)

**Supplementary Methods**

*Data collection*

We analysed a large sample of online videos from YouTube, using search terms including common great ape designations, such as, orangutan, gorilla, ape, AND rotational movements, such as, spinning, rolling, flipping. Descriptive statistics are provided in Supplementary Data. For replicability purposes, these terms can be used to generate an equivalent (and even potentially larger) data set of similar videos.

We identified 40 videos involving rotation sequences with 2 or more revolutions (i.e., spinning bouts of 1 revolution were not considered. Videos covered all great ape genera (orangutans, gorillas and chimpanzees and bonobos) and occurred at different sites (please see links to all identified and analysed videos below; in case of removed videos or broken links, access to a copy may be requested from the authors for non-distributive and educational purposes in accordance with copyright).

For ape-human comparison, instances of human ballet pirouettes, Ukrainian Hopak pirouettes, circus spinning rope acts, and Sufi Whirling dervishes were also selected from YouTube. To quantify rotational speeds across great apes and humans, and to code other features of the behaviours, we used QuickTime Player (Version 10.5). The ‘Trim’ function enabled frame-by-frame visual inspection to a precision of 1/100^th^ of a second, which we rounded to the nearest 1/10^th^ in our measurements.

*Data analyses* – *Rotational speed*

To compare rotational speed among great apes and investigate its behavioural correlates, we conducted linear mixed models using JASP (version 0.16.2)^1^ and Satterthwaite test model, type III sum of squares, two-sided. Rotational speed per revolution was inserted as dependent variable. Genus (three levels: *Pongo*, *Gorilla*, *Pan*), ground contact (three levels: none, light, heavy), end bout dismount (two levels: maintain holding onto rope, let go of rope/let rope go slack), number of grips, and total number of spins per bout were inserted as fixed effects. Spinning bout and video source were inserted as random effects to control for the effect of different bouts contributing different numbers of revolutions and of different clips contributing different numbers of spinning bouts. Model fit statistics, samples sizes and fixed effects estimates are provided in Supplementary Data.

To compare rotational speed between great apes and humans, we conducted a similar linear mixed model with taxon (two levels: great apes, humans) inserted as fixed effect, and spinning bout and video source inserted as random effects. No differences in rotational speed were *a priori* predicted to occur between different age and/or sex classes of the same species due to similar anatomy and similar underlying neurophysiology, so we opted not keep this variable out from our models to preserve statistical power. Model fit statistics, samples sizes and fixed effects estimates are provided in Supplementary Data.

To compare rotational speed between great apes and each human professional dance or spinning tradition, we conducted a similar linear mixed model with style (five levels: great apes, Sufi whirling, ballet pirouettes, circus spinning rope act, Hopak pirouettes) inserted as fixed effect and, spinning bout and video source inserted as random effects. Model fit statistics, samples sizes and fixed effects estimates are provided in Supplementary Data.

*Data analyses* – *Total number of revolutions per bout*

To compare total number of revolutions per bout among great apes and investigate its behavioural correlates, we conducted a similar linear mixed model with genus (three levels: *Pongo*, *Gorilla*, *Pan*), ground contact (three levels: none, light, heavy), end bout dismount (two levels: maintain holding onto rope, let go of rope/let rope go slack) and number of grips holds on the rope (up to 5, summing left/right hand, left/right foot, and/or mouth) inserted as fixed effects, and video source inserted as random effect. No differences in rotational speed were *a priori* predicted to occur between different age and/or sex classes of the same species due to similar anatomy and similar underlying neurophysiology, so we opted not keep this variable out from our models. Model fit statistics, samples sizes and fixed effects estimates are provided in Supplementary Data.

All figures were produced using R^2^ and the package ggplot2^3^.

**Links to all original video sources**

*Orangutans (Pongo)*

https://www.youtube.com/watch?v=ATxqFJQ2yIc

http://www.youtube.com/watch?v=MXddkEKNAic

http://www.youtube.com/watch?v=mkXPGmCngJ4

https://www.youtube.com/watch?v=xHMr65ViOPs

https://www.youtube.com/watch?v=pBbU1sozWHk

https://www.youtube.com/watch?v=dX_0lJER_oM

https://www.youtube.com/watch?v=KWF8r-M0LOE

https://www.youtube.com/watch?v=pIZm-bl5578

https://www.youtube.com/watch?v=QBlc9fYQYSM

http://www.youtube.com/watch?v=XiTaIbocM7k

https://www.youtube.com/watch?v=_lthBmJ7QxQ

https://www.youtube.com/watch?v=_Gg4gkMz2i8

http://www.youtube.com/watch?v=pVpvO7Bp4Fg

http://www.youtube.com/watch?v=dYXRMRvH6lU

http://www.youtube.com/watch?v=44WzNVi7xsQ

https://www.youtube.com/watch?v=Oy8ZGpnUP0g

https://www.youtube.com/watch?v=5ufrWqcJXkM

https://www.youtube.com/watch?v=l2C7Pv1yeZc

http://www.youtube.com/watch?v=DZBLVTovYCA

*Gorillas (Gorilla)*

https://www.youtube.com/watch?v=KfS07LkqniY

https://www.youtube.com/watch?v=Q8GQb1qiUtg

https://www.youtube.com/watch?v=45X_659xj2A

https://www.youtube.com/watch?v=8uEgybXoiKo

https://www.youtube.com/watch?v=45CBtlhABXo

http://www.youtube.com/watch?v=wf8-npgVkN0

https://www.youtube.com/watch?v=1T8_aDOkcJI

https://www.youtube.com/watch?v=gqnKSi4Hizk

https://www.youtube.com/watch?v=hZHLFwg7CRY

https://www.youtube.com/watch?v=Z7k6yqSWXWo

https://www.youtube.com/watch?v=Gueu4lBdJsY

https://www.youtube.com/watch?v=7ZuLf7jOJkg

https://www.youtube.com/watch?v=A6E1WpaldRc

https://www.youtube.com/watch?v=8wqixRn2Heg

*Chimpanzees and bonobos (Pan)*

http://www.youtube.com/watch?v=J19RDBMAOKU

https://www.youtube.com/watch?v=hjhdbHErxWQ

http://www.youtube.com/watch?v=KyOZ99bhs7k

http://www.youtube.com/watch?v=dLCFxfX4eg4

https://www.youtube.com/watch?v=BsoSGW-TWzQ

https://www.youtube.com/watch?v=JNXfG1IS-5c

https://www.youtube.com/watch?v=bbWzNT74q6E

*Humans (Homo)*

https://www.youtube.com/watch?v=zvD21OntHgI

https://www.youtube.com/watch?v=Q-Y7oKPdV70

https://www.youtube.com/watch?v=lFIQMM8bZQk

https://www.youtube.com/watch?v=y1XlyMnjGr0

https://www.youtube.com/watch?v=voIK5LhdGl4

<https://www.youtube.com/watch?v=m0jIgrQBxSM>

**References**

1. JAST Team (2020). JASP (University of Amsterdam).

2. Team, R. (2013). R: A language and environment for statistical computing.

3. Wickham, H. (2009). ggplot2: Elegant Graphics for Data Analysis (Springer-Verlag).
